# Investigation of Intervention Solutions to Enhance Adherence to Oral Anticancer Medicines in Adults: Overview of Reviews

**DOI:** 10.2196/34833

**Published:** 2022-04-27

**Authors:** Thu Ha Dang, Abdur Rahim Mohammad Forkan, Nilmini Wickramasinghe, Prem Prakash Jayaraman, Marliese Alexander, Kate Burbury, Penelope Schofield

**Affiliations:** 1 Department of Psychology School of Health Sciences Swinburne University of Technology Melbourne Australia; 2 Behavioural Sciences Unit Department of Health Services Research Peter MacCallum Cancer Centre Melbourne Australia; 3 Digital Health Cooperative Research Centre Sydney Australia; 4 Digital Innovation Lab School of Science, Computing and Engineering Technologies Swinburne University of Technology Melbourne Australia; 5 Department Health and Bio Statistics School of Health Sciences and Iverson Health Innovation Research Institute Swinburne University of Technology Melbourne Australia; 6 Epworth Healthcare Melbourne Australia; 7 Pharmacy Department Peter MacCallum Cancer Centre Melbourne Australia; 8 Sir Peter MacCallum Department of Oncology The University of Melbourne Melbourne Australia; 9 Digital and Healthcare Innovation Peter McCallum Cancer Centre Melbourne Australia; 10 Department of Psychology, and Iverson Health Innovation Research Institute Swinburne University of Technology Melbourne Australia

**Keywords:** digital, intervention, medication adherence, oncology, oral anticancer, systematic review

## Abstract

**Background:**

Adherence to anticancer medicines is critical for the success of cancer treatments; however, nonadherence remains challenging, and there is limited evidence of interventions to improve adherence to medicines in patients with cancer.

**Objective:**

This overview of reviews aimed to identify and summarize available reviews of interventions to improve adherence to oral anticancer medicines in adult cancer survivors.

**Methods:**

A comprehensive search of 7 electronic databases was conducted by 2 reviewers who independently conducted the study selection, quality assessment using the A Measurement Tool to Assess Systematic Reviews 2, and data extraction. The PRISMA (Preferred Reporting Items for Systematic Reviews and Meta-Analyses) 2020 checklist was adapted to report the results.

**Results:**

A total of 29 reviews were included in the narrative synthesis. The overall quality of the systematic reviews was low. The 4 main strategies to promote adherence were focused on education, reminders, behavior and monitoring, and multicomponent approaches. Digital technology–based interventions were reported in most reviews (27/29, 93%). A few interventions applied theories (10/29, 34%), design frameworks (2/29, 7%), or engaged stakeholders (1/29, 3%) in the development processes. The effectiveness of interventions was inconsistent between and within reviews. However, interventions using multiple strategies to promote adherence were more likely to be effective than single-strategy interventions (12/29, 41% reviews). Unidirectional communication (7/29, 24% reviews) and technology alone (11/29, 38% reviews) were not sufficient to demonstrate improvement in adherence outcomes. Nurses and pharmacists played a critical role in promoting patient adherence to oral cancer therapies, especially with the support of digital technologies (7/29, 24% reviews).

**Conclusions:**

Multicomponent interventions are potentially effective in promoting patient adherence to oral anticancer medicines. The seamless integration of digital solutions with direct clinical contacts is likely to be effective in promoting adherence. Future research for developing comprehensive digital adherence interventions should be evidence-based, theory-based, and rigorously evaluated.

## Introduction

With the advent of oral anticancer medicines (OACMs) more than 2 decades ago [1], there has been a gradual shift for cancer treatments to be increasingly administered at home [2]. Oncology care teams and their patients face new challenges in ensuring optimal adherence to therapy. Studies have revealed that the rate of adherence to OACMs varies widely across cancers, but it can be as low as 16% [3] and often worsens over time [4]. Medication adherence (MA) is defined as “the extent to which patients take their medication as recommended by their health care provider” [5]. Adherence is an important predictive factor for the success of OACMs [1,6], particularly when these therapies require patients to take medications correctly over a long period.

Given the high priority of adherence to OACMs in cancer care, there have been an increasing number of interventions to address MA issues, particularly in oral endocrine therapy for breast cancer [7] and oral medications for hematologic malignancies [8]. However, published reviews have disclosed that the evidence for these interventions is limited in both quantity [9] and quality [2].

In recent years, in an effort to provide more evidence in this area, there have been quite a few published reviews of adherence interventions in oncology, especially digital solutions [10-13]. However, these reviews varied in scope, methodology, and outcome of interest, which could overwhelm decision makers. This overview of reviews aimed to identify and summarize the available reviews of interventions to improve adherence to OACMs in adults with cancer. Overviews are new methodological approaches that have been used where multiple reviews already exist on the topic of interest to filter the plethora of information and provide a framework for clinical decision makers [14,15].

## Methods

### Overview

The study protocol was registered in the PROSPERO (International Prospective Register of Systematic Reviews) database (CRD42021240578) [16]. The PRISMA (Preferred Reporting Items for Systematic Reviews and Meta-Analyses) 2020 statement was adapted to report this systematic review of reviews [17] and is presented in Multimedia Appendix 1 [17].

### Search Strategy

A systematic literature search was performed on 7 databases for all publications up to March 2021: Ovid MEDLINE, Ovid Embase, CINAHL, PsycINFO, Web of Science, the Cochrane Database of Systematic Reviews, and the Database of Abstracts of Reviews of Effects. The Peer Review of Electronic Search Strategies checklist [18] was used to guide the development of the search strategy. The electronic search strategy was initially developed in MEDLINE by a reviewer (THD) and was then peer-reviewed by a group of experts in relevant fields (KB, MA, NW, PPJ, and PS) and a librarian to ensure its comprehensiveness. The search strategy combined controlled vocabulary and keywords, including synonyms, antonyms, and acronyms related to adherence, intervention, and cancer, and was adapted for each database. We did not limit the publication date but limited the search to the English language, human studies, and reviews only (refer to Multimedia Appendix 2 for full search strategies).

In addition to the database search, bibliographies of selected studies were also hand-searched to identify relevant studies not detected by the electronic search.

### Criteria for Considering Studies for This Review

The studies had to meet all the following criteria to be eligible for inclusion:

- Population: adults (≥18 years) diagnosed with any type of cancer undergoing OACMs. Studies on children were excluded because of the specificity of treatment issues in this group. Studies in a group of the population that separately reported results for adults with cancer were also included;
- Intervention: any type of intervention that included a component to enhance patient adherence to oncology treatment;
- Comparator: usual care or active control intervention;
- Outcome: MA compliance or persistence, clinical outcomes, and quality of life of people with cancer;
- Study type: reviews, including literature review or narrative review, scoping review, and systematic review.

### Study Selection

One reviewer (THD) conducted the searching, deduplication, and initial screening of titles and abstracts of all studies found. A second reviewer (ARMF) conducted a random independent assessment of the identified papers and reviewed the screening results of the first reviewer. Two reviewers independently screened all full texts of potentially eligible papers. When necessary, any differences between the 2 reviewers were discussed until consensus was reached or resolved by a third reviewer. Covidence site, operated by Veritas Health Innovation Ltd [19], was used for data screening, selection, and management.

### Assessment of Methodological Study Quality

The methodological quality of the included systematic reviews was independently assessed by 2 reviewers (THD and ARMF), adapting the A Measurement Tool to Assess Systematic Reviews (AMSTAR) 2, which has demonstrated satisfactory reliability and construct validity [20]. AMSTAR 2 is a tool used to evaluate the methodological quality of systematic reviews, which includes randomized and nonrandomized studies of interventions, including 10 domains and 16 items or questions. The answering options were *yes*, partial *yes*, or *no/no* information (corresponding to low or high risk of bias). We used the findings from the AMSTAR 2 critical appraisal to understand the certainty of the evidence base of the systematic reviews. Disagreements were resolved by discussion.

As AMSTAR 2 does not combine individual item ratings to create an overall score, the scheme for interpreting weaknesses detected in critical (7) and noncritical (9) items, proposed by Shea et al [20], was applied. The overall confidence in the results of the review was classified as *high*, *moderate*, *low*, or *critically low*, according to the number of critical and noncritical weaknesses identified in the systematic review under appraisal.

### Data Extraction and Synthesis

Data were independently extracted by 2 reviewers (THD and ARMF) in a standardized table (Multimedia Appendix 3), which was pilot-tested, for 7 random eligible studies, and then, results were compared and agreed upon. THD extracted data for the remaining eligible studies, which were then reviewed by ARMF, with discrepancies resolved through consensus. The corresponding authors of the included studies were contacted for further information or clarification, if necessary. The extracted data included the type of review, research questions, type of interventions, search strategies, search period limits, characteristics of included studies, quality assessments, methods of analyses, and findings. The included reviews were expected to have high heterogeneity in terms of interventions, comparators, outcome measures, study populations, and methodologies. Therefore, statistical pooling through meta-analysis was not appropriate.

## Results

### Overview

The results of this review are presented in the following order: search results, characteristics of included reviews, quality of systematic reviews, description of interventions, and outcomes of included reviews by group—scoping, systematic, and literature reviews. Owing to the heterogeneity of the included reviews, the findings are presented in a narrative format and refer to meta-analyses performed by the authors of the included reviews whenever available.

### Search Results

**Figure 1:**
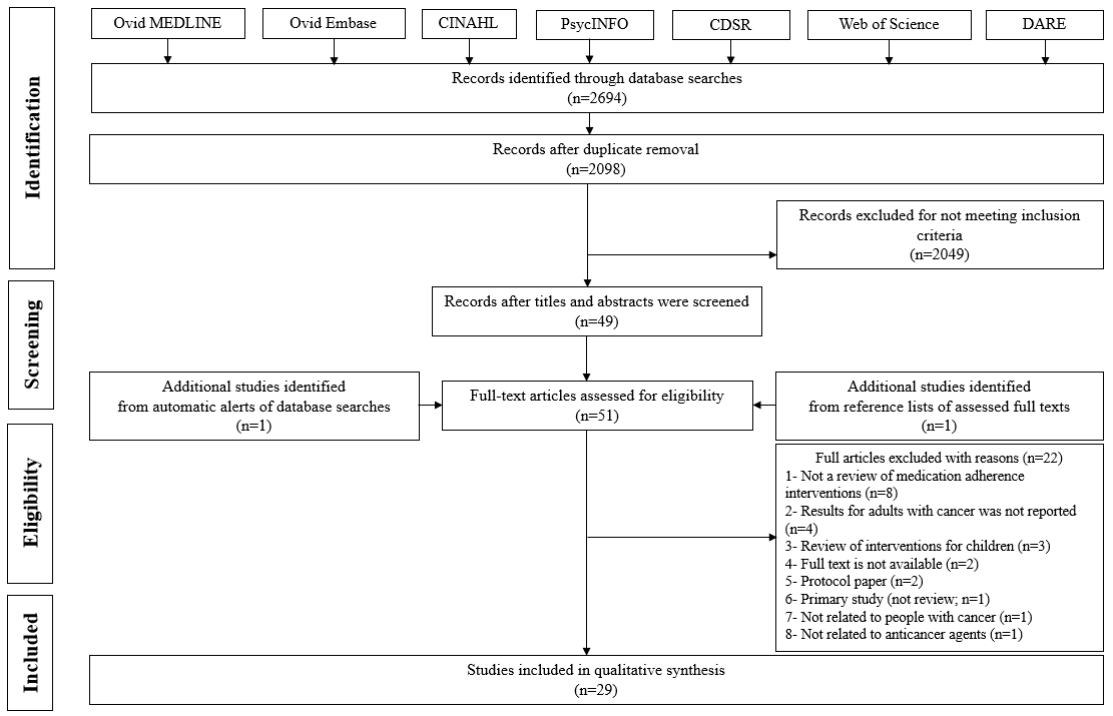
Flow diagram of study selection. CDSR: Cochrane Database of Systematic Reviews; DARE: Database of Abstracts and Reviews of Effects.

The search strategy identified 2098 unique results from 7 databases, 1 from the reference lists of the included studies, and 1 from the automatic alerts of the databases. Title and abstract screening identified 51 studies for full-text screening, of which 29 (57%) met the inclusion criteria. Details of the excluded studies and reasons for exclusion are provided in Multimedia Appendix 4 [2,7-9,12,21-46]. A high level of concordance was achieved between the 2 reviewers in the screening process, with disagreement in only 10% (5/51) of cases. These 5 papers were discussed by the 2 reviewers, and consensus was achieved. The selection process is illustrated in the flowchart (Figure 1).

### Characteristics of the Included Reviews

#### Study Design and Publication Time

Among the 29 included reviews, 12 (41%) were systematic reviews [2,7-9,12,21-27], 5 (17%) were scoping reviews [11,13,47-49], and the remaining 12 (41%) were literature reviews [10,28,29,50-58]. All 29 reviews were descriptive, with only 1 (3%) including meta-analyses [25]. All the studies were published in English. Of these 29 reviews, 25 (86%) were published between 2014 and 2021 and the remaining 4 (14%) were published before 2014 [28,50-52]. Although literature reviews were published throughout the period from 2009 to 2021, the results of all systematic reviews were publicly reported between 2014 and 2019, and the publication of 5 scoping reviews began in 2018.

#### Participants

Most reviews included studies on all types of cancer (22/29, 76%) [2,9-11,13,21,22,24,26-29,47-55,58], followed by breast cancer (6/29, 21%) [7,12,23,25,56,57], and hematological cancer (1/29, 3%) [8]. A total of 90% (26/29) reviews [2,7-13,21,23-29,48-57] examined adherence interventions for disease-modifying therapies, and 10% (3/29) reviews [22,47,58] reported adherence interventions for all types of cancer treatments. In total, 17% (5/29) reviews specifically focused on women [7,12,23,25,56], 7% (2/29) on adolescents and young adults [22,58], and 3% (1/29) on socially disadvantaged people with cancer [26]. The characteristics of the 29 studies included in this overview are presented in Multimedia Appendix 5.

#### Aims of the Reviews

Although all 29 reviews aimed to synthesize evidence of interventions used to promote MA among people with cancer, 6 (21%) narrative reviews also included available literature on adherence to oral anticancer regimens [2,28,50,52,53,58]. Of the 5 scoping reviews, 4 (80%) targeted digital adherence solutions, such as mobile apps [47,49], mobile phone–delivered interventions [48], and digital interventions in general [11]. Of the 12 systematic reviews, 6 (50%) focused on examining either the efficacy [22] or the effectiveness of adherence interventions [9,12,21,24,25]. Some of the reviews specifically focused on the type of interventions (eg, nurse-led [51], pharmacist-led [24], educational [21], and technology-mediated [10,11,47-49,54,55,57]), specific settings (eg, ambulatory care setting [21]), and socially disadvantaged groups in the Organization for Economic Co-operation and Development countries [26].

### Quality of Systematic Reviews

Among the 29 reviews, 12 (41%) were systematic reviews, of which only 1 (3%) conducted meta-analyses (Multimedia Appendix 5). Methodological quality was low or critically low overall, with at least 2 out of 16 AMSTAR 2 appraisal items [20] not met in all systematic reviews. The quality assessment of the 7 critical AMSTAR 2 domains is presented in Multimedia Appendix 6. Only 8% (1/12) systematic reviews from the study by Arthurs et al [21] received moderate overall confidence ratings in the reported results, which meant that this systematic review may provide an accurate summary of the results of the included studies to address the questions of interest. Moreover, 33% (4/12) and 59% (7/12) of systematic reviews received low and critically low overall confidence ratings, respectively, meaning that the summarized results of these studies may be inaccurate and that the conclusions need to be interpreted carefully. The best adherence was found for using the components of the PICO (population, intervention, comparison, and outcome) framework when describing the search question and inclusion criteria (item 1) and describing the included studies in adequate detail (item 8). The item that most reviews (9/12, 75%) failed to meet was providing a justification for excluding individual studies (item 7). For the critical domains, 42% (5/12) reviews referred to a review protocol (item 2), and 25% (3/12) reviews provided a list of excluded studies and justified their exclusion (item 7). Nearly all reviews (11/12, 92%) accounted for risk of bias when interpreting the results (item 13). Most of the reviews (10/12, 83%) used a satisfactory technique for assessing the risk of bias in individual studies (item 9), and 75% (9/12) of reviews conducted a comprehensive literature search (item 4). The only review with meta-analyses adhered to the item of using appropriate methods for statistical combination of the results (item 11) and investigated publication bias (item 15). More details on the bias assessments of all 16 AMSTAR 2 items are provided in Multimedia Appendix 7 [2,7-9,12,21-27].

### Description of Interventions

#### Overview

Given the wide range of aims mentioned above, interventions were categorized differently across and within reviews. Most reviews (21/29, 72%) reported diverse and multimodal interventions [2,7-9,12,13,21-29,50-54,56,58]; however, 28% (8/29) of reviews provided detailed technology-mediated interventions [10,11,22,47-49,55,57].

#### Modes of Delivery

Owing to the heterogeneity and lack of a common approach to categorizing the interventions in the reviews, we describe the modes of delivery for each one in Multimedia Appendix 8. Although interventions could be broadly classified as face-to-face or remote, these categories should only be considered as a guide because they were not always exclusive, owing to the complexity of interventions. For example, the same educational elements could be delivered via direct contact and web-based channels.

##### Face-to-face Interventions Only

The only 2 reviews in this group of interventions [50,51] were published the earliest among the reviews included in this study. One review [51] focused particularly on nurse-delivered interventions.

##### Remote Interventions Only

A total of 28% (8/29) of reviews reported only on nonface-to-face interventions with the assistance of technologies [10,11,22,47-49,55,57]. All these reviews were published in the last 6 years. Most were directed at individuals through various delivery modes, including phone, SMS text messages, and mobile apps.

##### Combined Face-to-face and Remote Interventions

A total of 66% (19/29) of reviews were concerned with either face-to-face or remote modes of delivery or complex multimodal interventions [2,7-9,12,13,21,23-29,52-54,56,58]. Interventions in these reviews were either single or multicomponent, often including education; reminders; and affective components, such as patient navigators, emotional and self-management support, and problem solving.

More details about interventions in each review are presented in Multimedia Appendix 9 [2,7-13,21-29,47-58].

#### Theoretical Frameworks

Only 34% (10/29) of reviews reported on theoretical frameworks. The most common theories were the Health Belief Model [59] and its subsequent versions, Social Learning Theory, and Social Cognitive Theory [60], which were mentioned in 21% (6/29) of reviews [12,13,22,23,28,48]. The Self-Regulation Model [61] was the second most common framework, featured in 10% (3/29) of reviews [13,22,49]. One review [48] mentioned self-determination theory [62]. None of the face-to-face intervention reviews discussed theoretical frameworks.

#### Intervention Providers

As interventions are diverse, their providers include a range of professionals in the health care field: clinicians, nurses, pharmacists, and health providers. The interventions in most reviews were delivered by a multidisciplinary team. However, one review specifically focused on nurse-led interventions [28] and another on pharmacist-led interventions [24]. A total of 10% (3/29) of reviews reported on interventions delivered by nurses or pharmacists [21,27,51].

#### Intervention Development

Most reviews did not discuss the development of interventions. Using design frameworks and engaging stakeholders were rarely mentioned. One review [48] reported that stakeholders were engaged in the design of all included interventions, for example, patients and oncology clinicians were engaged in the early design phases to explore end users’ perceptions of the acceptability and usefulness of the interventions. Stakeholders were patients, caregivers, clinicians, administrators, care providers, the community, and society, depending on the type of intervention. Two design frameworks [63,64] were applied in the development of interventions in 2 reviews [48,49].

#### Dose and Duration

Although the doses and durations were mentioned in 31% (9/29) of reviews [7,10,13,21,24,29,48,53,57], they were brief and varied for different types of interventions and modes of delivery. For example, the frequency of SMS text messages was daily, bidaily, or weekly [7,10,29,48,57]. Automated voice responses could be set up on a daily, weekly, or monthly basis [10,29]. The duration of multicomponent interventions varied from 9 to 18 months [29]. The follow-up period of interventions could be as short as 2 months or as long as 45 months [24].

### Outcomes of Included Reviews by Group

#### Overview

All reviews, except 3 [47,49,51], reported MA improvement as a primary outcome. Some also reported medication persistence [23]; clinical outcomes, such as symptoms and adverse events [24]; hospital admission rates [9]; subclinical responses; survival time [8]; cancer-related knowledge and self-management skills [22,26]; and some quality-of-life indicators [26]. A total of 10% (3/29) studies [24,26,49] mentioned patient satisfaction and economic impact outcomes [24]. For this review, we focused on MA outcomes and discussed some of the secondary outcomes. The results from the 5 scoping reviews are described first, followed by 12 systematic reviews, and finally, the findings from the 12 narrative reviews.

#### MA (Primary Outcome)

##### Overview

Not all reviews specified how MA was measured. In reviews that specified how MA was measured, the methods were diverse: subjective, objective, or biomedical. Subjective measurements, such as patient self-reports and clinician reports, were the easiest reporting methods. However, perhaps because of its potential inaccuracy, it was only used to measure adherence in 12 reviews [2,7,8,10,12,13,21,23,25,52,53,56]. Half (14/29, 48%) of the reviews reported objective measurements, such as pill diaries, pill counts, and medication event monitoring systems [2,7-10,12,13,21,23,25,28,51-53], whereas some (7/29, 24%) mentioned biomedical measurements, such as drug metabolites in urine [2,7-10,52,56].

##### Scoping Reviews

MA was reported as a primary outcome in 60% (3/5) of scoping reviews [11,13,48] (Multimedia Appendix 10). Skrabal Ross et al [48] explored the evidence of mobile-delivered interventions, mainly SMS text messages and mobile apps (5 studies). Gambalunga et al [11] focused on mobile apps (7 studies). Both reviews concluded that despite the use of digital means in facilitating the adherence of patients with cancer to oral treatments being strongly recognized in the literature, its effectiveness was either underexamined [48] or poorly supported [11]. The engagement of stakeholders and the use of design frameworks in developing digital interventions were very important [48]. In a scoping review of 56 studies evaluating adherence to oral antineoplastic agents [13], less than half (n=25, 45%) reported statistically significant improvements in adherence or persistence. Of these 56 studies, 8 (14%) used a mobile health tool and SMS text messages as the mode of delivery. The results revealed that drug-reminder SMS text messaging, either alone or in combination with a mobile app targeting intentional nonadherence, appeared to be effective among people with a single diagnosis but not among those with different diagnoses. The review also emphasized that theory-based and evidence-based interventions tailored to the needs of patients were more likely to be effective.

In the other 2 scoping reviews [47,49], mobile apps were reported as useful tools in facilitating the delivery of behavioral guidance, real-time capture of patients’ symptoms, monitoring of adherence, and supporting the self-management of side effects [49]. Nevertheless, the efficacy of mobile apps in improving symptom management and MA requires further exploration [48].

##### Systematic Reviews

MA was the primary outcome of all 12 systematic reviews. Findings from the meta-analytic results are presented first, followed by narrative syntheses.

Only 1 systematic review by Finitsis and Vose [25] contained meta-analyses to quantify the aggregate effect of interventions to improve adjuvant endocrine therapy adherence among women with breast cancer and meta-analyzed these effects across studies. A total of 7 studies that reported 8 interventions were included in this review [30-35,65]. Nearly half (3/7, 43%) of the included studies used one-way communication to deliver information and education to patients. Two studies used bidirectional communication between oncology nurses and patients. One study used a multicomponent intervention, including a mobile app and phone call follow-up from the care team. The results showed that interventions using bidirectional communication (ie, eliciting information from patients and sending information to patients) had statistically significant effects compared with the control groups within each study (*k*=4; Cohen *d*=0.59; 95% CI 0.23-0.95), whereas those using only one-way communication (ie, purely providing information to patients) did not (*k*=4; Cohen *d*=−0.03; 95% CI −0.27 to 0.20). The authors concluded that the interventions failed when one-way flow communication was used. Interventions to improve adjuvant endocrine therapy adherence should enhance patient engagement via bidirectional platforms. The additional details are presented in Multimedia Appendix 11 [13-19].

MA was reported as a primary outcome in all 11 narrative systematic reviews [2,7-9,12,21-24,26,27]. Four main strategies to promote adherence emerged from these reviews: education, reminders, behavior and monitoring, and multicomponent interventions. The reported results varied between and within reviews, even for the same types of intervention (Multimedia Appendix 12).

The educational strategy was reported in all reviews, either as a stand-alone intervention or as an element of multicomponent interventions. Educational materials often included information about diseases and medications (eg, dosage, side effects, storage, disposal, and ways to remember to take the medication). Studies revealed that education alone, regardless of delivery (eg, face-to-face, leaflets, or mailouts), was insufficient to promote adherence to anticancer regimens [2,7,8,12,23,27].

There are many mechanisms that can be used to remind patients to take their medication. These could be as simple as calendars, diaries, dosing sheets, pillboxes, and charts or more advanced, with the help of technology, such as SMS text messages and mobile apps. Although reminders could be effective in reinforcing the behavior of taking the medication in some chronic conditions, such as HIV or AIDS [52,53], their effectiveness in oncology has not been demonstrated [7,9,26].

The behavioral and monitoring strategies have been broadly used in MA interventions in various forms and modes of delivery: delivered either in a single form or mode (monitoring pill-taking, autopharmacy refills, electronic prescribing, and individual coaching) [2,7,23,24] or an intervention package (monitoring and feedback, side effect management, and positive self-care behavior) [2,7,22,23]. Similar to the diversity of interventions within this group of strategies, their effectiveness in enhancing adherence to oral antineoplastic medicines varied widely within and between reviews [2,7,9,22,26,27].

Multicomponent interventions were reported in 82% (9/11) of systematic reviews [2,7-9,12,21,23,24,26], often including a combination of education; reminders; and behavioral, cognitive, or affective components. Tailored education in combination with drug reminders and counseling delivered by nurses or pharmacists to promote symptom management and adherence behavior was likely to be effective in improving adherence [2,8,9,12,24]. Nevertheless, in a few (4/29, 14%) reviews, nurse-led tailored patient education [21], pharmacist-led intensive care programs [21], and education combined with reminder interventions were not effective [7,23]. The effect of education, pill shaping, and home restructuring was uncertain in the systematic review by Mathes and Antoine [9]. This uncertainty was also observed in multicomponent interventions including education, reminders, and motivational interviewing [23]; or interventions including education and monitoring [27].

There were some overlaps across systematic reviews at the individual-study level. The results of 18 primary studies, including 7 randomized controlled trials [30,34-37,66,67], were reported in more than 1 systematic review [30,33-44,65-69]. For example, 2 randomized controlled trials on the compliance of patients to anastrozole in a therapy program, published by Hadji et al [66], and the influence of a patient information program on adherence and persistence to an aromatase inhibitor in breast cancer treatment, published by Ziller et al [35], were reported in the same 5 systematic reviews [2,7,12,23,25]. More details on the overlap of primary studies across systematic reviews are presented in Multimedia Appendix 13 [13,15-27,47-50].

##### Literature Reviews

Among the 12 included literature reviews, 4 (33%) focused on technology-based interventions; 1 (8%) examined nursing interventions [51]; and 2 (17%) expanded the scope of research to areas such as adherence or persistence rates [50] and its impacts [53], challenges to adherence in oncology [28,52,53], and adherence measurements [52]. MA was reported as a primary outcome in all literature reviews except 2 [51,57] (Multimedia Appendix 14).

The results from these literature reviews were consistent with findings from included scoping and systematic reviews: education alone was insufficient to promote adherence to oral medication regimens [29,53,54,58], and multicomponent interventions were more likely to be effective in improving adherence [10,28,29,50,52-55,58]. Behavioral and monitoring strategies did not consistently improve adherence rates when used alone [29,50,53], although some studies have reported positive results [28,29,56].

However, the effectiveness of reminders was controversial. Reminder tools, such as calendars, diaries, and dosing sheets, likely improved patient adherence [28,53], whereas daily pillboxes were unlikely to do so [28,50]. Electronic reminders, such as SMS text messages and mobile apps, were reported to be effective in the review of Accordino and Hershman [52] but ineffective in another review conducted by Cazeau [10].

Narrative reviews also revealed that oncology nurses and pharmacists, as part of a multidisciplinary team, can have a significant influence on patient adherence via education, increased access to medicines, early identification of symptoms, and side effect self-management skills [28,29,51,53].

#### Secondary Outcomes

In addition to MA rates, clinical outcomes, such as decreased symptoms [24], cytogenetic response, and survival time [8], were evaluated. The effects of education on clinical outcomes were uncertain [8]. However, some multicomponent interventions, including education, tailored counseling, and affective components (eg, home visit support), showed possible positive effects [8,24]. In 2 reviews [22,26], interventions combining side effect management, positive self-care behavioral promotion, education, counseling, or organizational change elements improved cancer-related knowledge and self-efficacy among people with cancer. Two reviews [9,24] listed hospital admission rate as a secondary outcome, but it was not statistically significant in all included interventions.

## Discussion

### Principal Findings

This overview of reviews aimed to synthesize evidence from available reviews on interventions to improve MA to OACMs in adults with cancer. To the best of our knowledge, this is the first study to achieve this goal. Among the 29 included reviews, only 1 (3%) conducted meta-analyses and 17 (59%) did not follow systematic methodologies in identifying, analyzing, and reporting literature. Consequently, it was impossible to perform quantitative analyses. Nevertheless, including literature reviews in the narrative synthesis is useful for understanding the breadth of the study field. The only systematic reviews of moderate quality focused on therapeutic patient education interventions in ambulatory care settings [21]. The other 11 systematic reviews on the topic of interest had low or critically low confidence rating. Therefore, the results of the included reviews should be interpreted with caution.

The comparability of the study results is limited because of the high heterogeneity of the included reviews (Multimedia Appendix 5) and studies within each review [9,13]. The content of adherence-enhancing interventions is varied [29]. In addition, there are differences in the characteristics of patients whose adherence has been influenced [9]. Furthermore, comparability is constrained owing to different adherence measurements [2,7,8,52]. Accordingly, this review summarizes the main themes of the included reviews rather than comparing them.

This review suggests that single strategies to promote adherence (eg, education, reminders, or monitoring) are not sufficient to improve adherence. Multidimensional interventions that used collective strategies to promote adherence (education, reminder, cognitive, behavioral, and affective) were potentially more effective. Our findings are in line with earlier reviews of interventions to improve adherence in various chronic conditions [45,46] and those focusing on cancer [9,50,54,58]. These findings also resonate with the report of the World Health Organization that MA is a multidimensional phenomenon determined by 5 dimensions (social and economic, health system, condition-related, therapy-related, and patient-related) [70]. Thus, multicomponent interventions applying different strategies are needed to address the multifaceted adherence phenomenon [70].

The described theoretical frameworks were neither clear nor validated. One-third of reviews reported on the scattered use of cognitive and behavioral theories in only a few studies [12]. Although the authors [12,48] emphasized the importance of using theoretical grounding in planning, designing, and evaluating outcomes of multilevel interventions, a few [13,23] argued that the effect of this was quite modest. This uncertainty is in line with a meta-analysis of 683 studies that quantified the impact of theory-driven interventions on adherence [71]. The limited use of theory to design interventions means that no conclusions can be drawn regarding the importance and effectiveness of theoretically derived interventions. Furthermore, the complex and multifaceted factors contributing to nonadherence represent another challenge in the selection of an appropriate conceptual model to design interventions. Perhaps, a combination of theories may better explain the diverse barriers and facilitators of MA and provide a stronger direction to formulate interventions. Future research should pay more attention to this aspect of adherence interventions.

The use of digital solutions to enhance adherence to cancer treatment has been increasing in the past decade [47,55]. The literature has emphasized the potential of digital platforms to facilitate oral antineoplastic adherence among people with cancer [11]. Medication nonadherence can be intentional or unintentional. Intentional nonadherence is a patient’s conscious decision not to take a drug, for example, because of unpleasant side effects [72]. Unintentional nonadherence is unplanned by a patient, for example, because of forgetfulness [73]. Therefore, the interventions require different modes of action. A variety of measures, such as patient education and good patient-provider communication, can enable patients to better report and manage therapeutic side effects [55]. Technologies can enhance these measures by providing patients with rapid, continuous, and easy access to both educational resources and symptom self-management strategies, also facilitating communication between patients and their care teams [11,55]. Personal lifestyle and electronic triggers (eg, SMS text messages) remind and motivate patients to take their medication, so that it becomes an integral part of their daily activities [11,52]. In both cases, digital platforms (eg, mobile apps) can enable real-time monitoring of patient self-management [11]. However, this is an emerging field, and most studies have focused only on evaluating the acceptability, usability, and feasibility of interventions. The effectiveness of digital MA interventions in clinical oncology practice is poorly supported [47,48]. Future research should not only focus on determining the effect of digital interventions on adherence but also on identifying barriers to delivering high-quality personalized care to end users [11].

Using frameworks and engaging stakeholders in the design and development of digital interventions is crucial. Design frameworks help in planning the resources needed for each stage of the design and to mobilize them effectively and efficiently [48]. The involvement of stakeholders is central in ensuring that the intervention meets the needs of the target audience and in increasing its sustainability [49]. Nevertheless, strategies involving stakeholders (eg, patients, caregivers, oncology clinicians, nurses, pharmacists, and the community) have rarely been reported [48]. The involvement of professionals in the intervention development processes was very limited [49,55]; only 2 studies [74,75] mentioned patients’ and clinicians’ participation. Most interventions did not use or, at least, did not report the use of design frameworks in the development processes [48]. Given the rapid increase of technology applications in MA and the importance of this aspect in intervention development, it is worthy of future research into the involvement of stakeholders and the design framework used in the development of adherence interventions.

The findings from this review show that the use of digital solutions alone may be insufficient and may require cultural adaptive change [57]. Health care professionals’ interaction with patients is pivotal to augmenting the effect of these interventions [51]. Nurses and pharmacists are uniquely positioned to promote adherence to oral cancer therapies [10,51,53]. Findings suggest that clinical support (eg, tailoring education to meet patients’ needs) and symptom assessment and management provided by nurses empowered patients’ ability to adhere to treatments [8,28,55]. Future interventions in cancer should maximize the advantage that health professionals can contribute to patients’ MA with the support of digital technology.

Finally, this review suggests that to consolidate evidence on the effects of MA interventions in cancer, further work is needed using rigorous methods, such as prospective randomized designs in large samples of patients. Study outcomes should not only be limited to adherence rates but also the long-term effects of interventions and meaningful clinical outcomes, such as decreased symptoms and adverse effects of therapy, inhibited disease progression, and increased patient survival and quality of life. These suggestions are consistent with the results of some other systematic reviews of interventions to promote adherence to OACMs that have been published to date [2,9].

### Limitations

This review has inevitable limitations owing to the limited existing high-quality quantitative analytic evidence, which also demonstrates a high risk of bias. Similarly, the significant heterogeneity across and within reviews and studies did not allow statistical analyses beyond reporting of results from the only meta-analysis and narrative analyses performed by the authors of the included reviews. Throughout the process, we relied on published evidence rather than aggregated data from individual studies. Therefore, a definitive assessment of the overall strength of evidence and the effectiveness of current interventions to enhance adherence to anticancer medicines among adults with cancer is not possible. Finally, only the reviews published in English were included. Thus, there is a risk of missing the relevant literature published in other languages. However, comprehensive searches were conducted using different databases to minimize this limitation as much as possible.

### Conclusions

Despite these challenges, this review suggests the potential effectiveness of multicomponent interventions to promote adherence to OACMs in adults. This review highlights the role of digital health in enabling and enhancing multicomponent adherence interventions. Nurses and pharmacists are in unique positions and play an important role in facilitating and motivating patient adherence behavior in oncology treatments. These processes can be facilitated without creating a burden if they are integrated into the current routine practices with the support of technology. The findings from this review support the need for future research in developing evidence-based digital multicomponent interventions to assist people with cancer in adhering to their oral therapies. This review also underscores the importance of stakeholders’ involvement and the use of a design framework in the development of interventions to increase translatability and sustainability in real oncology practices. Given the rapidly increasing use of oral antineoplastic medicines and the dramatic availability of digital tools worldwide, research in this field is expected to increase rapidly.

## Appendix

Multimedia Appendix 1 PRISMA (Preferred Reporting Items for Systematic Reviews and Meta-Analyses) 2020 checklist.

Multimedia Appendix 2 Search strategy.

Multimedia Appendix 3 Data extraction table.

Multimedia Appendix 4 Table of excluded studies.

Multimedia Appendix 5 Characteristics of included reviews.

Multimedia Appendix 6 Methodological quality of included systematic reviews (A Measurement Tool to Assess Systematic Reviews 2 critical domains, adapted from Shea et al [20]).

Multimedia Appendix 7 Methodological quality of included systematic reviews.

Multimedia Appendix 8 Interventions grouped according to modes of delivery.

Multimedia Appendix 9 Intervention details of included reviews.

Multimedia Appendix 10 Outcome of included scoping reviews.

Multimedia Appendix 11 Outcomes of included systematic reviews with meta-analyses.

Multimedia Appendix 12 Outcomes of included systematic reviews—narrative reviews.

Multimedia Appendix 13 Primary studies overlap across systematic reviews.

Multimedia Appendix 14 Outcome of included literature reviews.

## Abbreviations

AMSTAR: A Measurement Tool to Assess Systematic Reviews
MA: medication adherence
OACM: oral anticancer medicine
PICO: population, intervention, comparison, and outcome
PRISMA: Preferred Reporting Items for Systematic Reviews and Meta-Analyses
PROSPERO: International Prospective Register of Systematic Reviews
