# Supplementary material for: Investigation of Intervention Solutions to Enhance Adherence to Oral Anticancer Medicines in Adults: Overview of Reviews
Source: JMIR Cancer. 2022 Apr 27;8(2):e34833. doi: 10.2196/34833 (PMC9096640; doi:10.2196/34833)
Supplement: Multimedia Appendix 2 [file cancer_v8i2e34833_app2.docx]

Appendix 2. Search strategy

**Ovid MEDLINE(R) ALL <1946 to March 01, 2021>**

1 Behavior Therapy/

2 Internet-Based Intervention/

3 Telemedicine/

4 Mobile Applications/

5 (intervention* or program* or "behavio* change*").tw,kf.

6 1 or 2 or 3 or 4 or 5

7 exp "Treatment Adherence and Compliance"/

8 ((medication* or drug* or treatment or therap* or patient*) adj2 (noncompli* or nonadher*)).tw,kf.

9 7 or 8

10 Neoplasms/

11 cancer*.tw,kf.

12 10 or 11

13 6 and 9 and 12

14 limit 13 to human

15 limit 14 to english

16 meta analysis.mp,pt. or review.pt. or search:.tw.

17 15 and 16

**Embase <1974 to 2021 March 01>**

1 behavior therapy/

2 web-based intervention/

3 telemedicine/

4 mobile application/

5 (intervention* or program* or "behavio* change*").tw,kw.

6 1 or 2 or 3 or 4 or 5

7 exp patient compliance/

8 exp medication adherence monitoring system/

9 ((medication* or drug* or treatment or therap* or patient*) adj2 (noncompli* or nonadher*)).tw,kw.

10 7 or 8 or 9

11 neoplasm/

12 cancer*.tw,kw.

13 11 or 12

14 6 and 10 and 13

15 limit 14 to human

16 limit 15 to english

17 meta-analys:.mp. or search:.tw. or review.pt.

18 16 and 17

**PsycINFO**

Results for **Any Field**: ((medication*) OR (drug*) OR (treatment) (therap*) OR (patient*)) AND ((adher*) OR (comply) OR (complian*) OR (noncompli*) OR (nonadher*)) *AND* ((**Any Field**: "Behavio* Therapy") *OR* (**Any Field**: Intervention) *OR* (**Any Field**: telemedicine) *OR* (**Any Field**: "mobile phones") *OR* (**Any Field**: "behavio* change") *OR* (**Any Field**: "mobile app*") *OR* (**Any Field**: "computer applications")) *AND* ((**Any Field**: neoplasms) *OR* (**Any Field**: cancer*)) *AND* **Methodology**: Literature Review *OR* Systematic Review *OR* Meta Analysis *OR* Metasynthesis

**CINAHL**

S1 ("Behavio#r Therapy" or telemedicine or intervention* or program* or "behavio* change*" or "mobile app*")

S2 ((medication* or drug* or treatment or therap* or patient*) n2 (adher* or comply or complian* or noncompli* or nonadher*))

S3 (cancer*)

S4 S1 AND S2 AND S3

S5 (S1 AND S2 AND S3) AND ((review or "systematic review" or meta-analysis or "literature review" or metasynthesis))

**Web of Sciences**

TOPIC: (((medication* or drug* or treatment or therap* or patient*) near/2 (adher* or comply or complian* or noncompli* or nonadher*))) AND TOPIC: (("Behavior Therapy" or "telemedicine" or intervention* or program* or "behavio* change*" or "mobile app*")) AND TOPIC: ((cancer*)) AND TOPIC: ((review or "systematic review" or "literature review" or meta-analysis or metasynthesis))

Refined by: LANGUAGES: ( ENGLISH )

Timespan: All years. Indexes: SCI-EXPANDED, SSCI, A&HCI, CPCI-S, CPCI-SSH, BKCI-S, BKCI-SSH, ESCI, CCR-EXPANDED, IC.

**Cochrane Database of Systematic Review (CDSR)**

ID Search Hits

#1 MeSH descriptor: [Behavior Therapy] this term only

#2 MeSH descriptor: [Internet-Based Intervention] this term only

#3 MeSH descriptor: [Telemedicine] this term only

#4 MeSH descriptor: [Mobile Applications] this term only

#5 (intervention* or program* or "behavio* change*")

#6 #1 or #2 or #3 or #4 or #5

#7 MeSH descriptor: [Treatment Adherence and Compliance] explode all trees

#8 ((medication* or drug* or treatment or therap* or patient*) near/2 (noncompli* or nonadher*))

#9 #7 or #8

#10 MeSH descriptor: [Neoplasms] this term only

#11 cancer*

#12 #10 or #11

#13 #6 and #9 and #12

**Database of Abstracts of Reviews of Effects (DARE)**

(treatment adherence or treatment compliance or medication adherence or medication compliance or treatment refusal or drug adherence or drug compliance) AND (intervention or telemedicine or mobile application* or program or behavio* change*) AND (neoplasms or cancer*)
