# Supplementary material for: Investigation of Intervention Solutions to Enhance Adherence to Oral Anticancer Medicines in Adults: Overview of Reviews
Source: JMIR Cancer. 2022 Apr 27;8(2):e34833. doi: 10.2196/34833 (PMC9096640; doi:10.2196/34833)
Supplement: Multimedia Appendix 4 [file cancer_v8i2e34833_app4.docx]

Appendix 4: Table of excluded studies and reasons for exclusion (n = 22)

| No | Year | Authors | Title | Reason for Exclusion |
| --- | --- | --- | --- | --- |
|  | 1989 | Ellen R. Gritz, M. Robin DiMatteo and Ron D. Hays  [1] | Methodological issues in adherence to cancer control regimens | This is not a review of medication adherence interventions |
|  | 1998 | Peterman, Amy H., Cella, David F.  [2] | Adherence issues among cancer patients | Full text is not available |
|  | 1998 | Debra L. Roter, Judith A. Hall, Rolande Merisca, Beth Nordstrom, Deborah Cretin and Bonnie Svarstad  [3] | Effectiveness of Interventions to Improve Patient Compliance: A Meta-Analysis | Not focus on cancer (only 8/153 of included studies were related to cancer) |
|  | 2005 | Macrodimitris, Sophia D. [4] | Interventions to promote treatment adherence in patients with chronic physical illnesses: A review | Full text is not available |
|  | 2013 | M. Bryant Howren, Julia R. Van Liew and Alan J. Christensen  [5] | Advances in patient adherence to medical treatment regimens: The emerging role of technology in adherence monitoring and management | The study population is not people with cancer |
|  | 2013 | SanSoucie, Holly  [6] | Patient adherence to oral oncolytics | This is not a review of medication adherence interventions |
|  | 2014 | Nieuwlaat R, Wilczynski N, Navarro T, Hobson N, Je ery R, Keepanasseril A, Agoritsas T, Mistry N, Iorio A, Jack S, Sivaramalingam B, Iserman E, Mustafa RA, Jedraszewski D, Cotoi C, Haynes RB  [7] | Interventions for enhancing medication adherence (Review) | This is not a review of interventions in cancer (only 1/182 of included studies was related to cancer) |
|  | 2015 | Niraj Mistry, Arun Keepanasseril, Nancy L. Wilczynski, Robby Nieuwlaat, Manthan Ravall, R. Brian Haynes, and the Patient Adherence Review Team  [8] | Technology-mediated interventions for enhancing medication adherence | This is not a review of interventions in cancer (only 1/38 of included studies was related to cancer) |
|  | 2017 | Sumit Gupta, Smita Bhatia  [9] | Optimizing medication adherence in children with cancer | The study population is not adult. |
|  | 2017 | Sarah S. Mougalian, Lianne N. Epstein, Ami P. Jhaveri, Gang Han, Maysa Abu-Khalaf, Erin W. Hofstatter, Michael P. DiGiovanna, Andrea L.M. Silber, Kerin Adelson, Lajos Pusztai, Cary P. Gross  [10] | Bidirectional Text Messaging to Monitor Endocrine Therapy Adherence and Patient-Reported Outcomes in Breast Cancer | This is a primary study (not a review) |
|  | 2017 | Teresa White, Heidi Larson, Alexandra Minnella and Howard S. Hochster  [11] | Metastatic colorectal cancer management with trifluridine/tipiracil | This is not a review of medication adherence interventions |
|  | 2018 | Wendy H. Oldenmenger, Jenske I. Geerling, Irina Mostovaya, Kris C.P. Vissers, Alexander de Graeff, Anna K.L. Reyners, Yvette M. van der Linden  [12] | A systematic review of the effectiveness of patient-based educational interventions to improve cancer-related pain | This is not a review of adherence interventions to anti-cancer agents |
|  | 2019 | Kelly D. Coyne, Katherine A. Trimble, Ashley Lloyd, Laura Petrando, Jennie Pentz, Andrea Fawcett, and Catherine M. Laing  [13] | Interventions to Promote Oral Medication Adherence in the Pediatric Chronic Illness Population: A Systematic Review From the Children’s Oncology Group | The study population is not adult. |
|  | 2019 | Flávia Oliveira Almeida Marques Cruz, Ricardo Alencar Vilela, Elaine Barros Ferreira, Nilce Santos Melo, Paula Elaine Diniz Dos Reis  [14] | Evidence on the Use of Mobile Apps During the Treatment of Breast Cancer: Systematic Review | This is not a review of medication adherence interventions |
|  | 2019 | Milena Dalton, Emily Holzman, Erica Erwin, Sophia Michelen, Anne F. Rositch, Somesh Kumar, Verna Vanderpuye, Karen Yeates, Erica J. Liebermann, Ophira Ginsburg  [15] | Patient navigation services for cancer care in low-and middle-income countries: A scoping review | This is not a review of medication adherence interventions |
|  | 2019 | Kleyton Santos Medeiros, Janice França Queiroz, Michelly Nóbrega Monteiro, Weruska Alcoforado Costa, Ricardo Ney Cobucci, Beatriz Stransky, Ana Katherine Gonçalves  [16] | Impact of mobile applications on adherence to cancer treatment: a systematic review and meta-analysis protocol | This is a protocol paper |
|  | 2020 | Carmen Chai Wang Er , Lau Bee Theng, Abdullah Al Mahmud, Mark Tee Kit Tsun  [17] | A Survey of Digital Health Interventions for Children with Cancer | The study population is not adult. |
|  | 2020 | Pinelopi Konstantinou, Angelos P. Kassianos, Giοrgos Georgiou, Andreas Panayides, Alexia Papag eorgiou, Ioannis Almas, Greta Wozniak, Maria Karekla  [18] | Barriers, facilitators, and interventions for medication adherence across chronic conditions with the highest non-adherence rates: a scoping review with recommendations for intervention development | Not focus on cancer (only 6/243 of included studies were related to cancer) |
|  | 2020 | Maria A. Lopez-Olivo, Kristin G. Maki, Noah J. Choi; Richard M. Hoffman, Ya-Chen Tina Shih, Lisa M. Lowenstein, Rachel S. Hicklen, Robert J. Volk  [19] | Patient Adherence to Screening for Lung Cancer in the US: A Systematic Review and Meta-analysis | This is not a review of medication adherence interventions |
|  | 2020 | Mostafa R. Mohamed, Erika Ramsdale, Kah Poh Loh, Asad Arastu, Huiwen Xu, Spencer Obrecht, Daniel Castillo, Manvi Sharma, Holly M. Holmes, Ginah Nightingale, Katherine M. Juba, Supriya G. Mohile  [20] | Associations of Polypharmacy and Inappropriate Medications with Adverse Outcomes in Older Adults with Cancer: A Systematic Review and Meta-Analysis | This is not a review of medication adherence interventions |
|  | 2020 | Melanie Murphy, Kathleen Bennett, Carmel M. Hughes, Amanda Lavan, Cathal A. Cadogan  [21] | Interventions to optimise medication prescribing and adherence in older people with cancer: A systematic scoping review | This is a protocol paper |
|  | 2020 | Shejila Chillakunnel Hussain Rawther, Mamatha Shivananda Pai, Donald J Fernandes, Stanley Mathew, Jyothi Chakrabarty, Elsa Sanatombi Devi  [22] | Specialist nurse initiated interventions in breast cancer care: A systematic review of randomised controlled trials | This is not a review of medication adherence interventions |

1. Gritz ER, DiMatteo MR, Hays RD. Methodological issues in adherence to cancer control regimens. Preventive Medicine. 1989 Sep;18(5):711-20. PMID: 2694165.

2. Peterman AH, Cella DF. Adherence issues among cancer patients. The handbook of health behavior change, 2nd ed. New York, NY, US: Springer Publishing Company; 1998. p. 462-82.

3. Roter DL, Hall JA, Merisca R, Nordstrom B, Cretin D, Svarstad B. Effectiveness of interventions to improve patient compliance - A meta-analysis. Med Care. 1998 Aug;36(8):1138-61. PMID: WOS:000075224200004. doi: 10.1097/00005650-199808000-00004.

4. Macrodimitris SD. Interventions to promote treatment adherence in patients with chronic physical illnesses: A review. Hellenic Journal of Psychology. 2005;2(2):115-34.

5. Howren MB, van Liew JR, Christensen AJ. Advances in patient adherence to medical treatment regimens: The emerging role of technology in adherence monitoring and management. Social and Personality Psychology Compass. 2013;7(7):427-43. doi: 10.1111/spc3.12033.

6. SanSoucie H. Patient adherence to oral oncolytics: Walden University; 2013.

7. Nieuwlaat R, Wilczynski N, Navarro T, Hobson N, Jeffery R, Keepanasseril A, et al. Interventions for enhancing medication adherence. Cochrane Db Syst Rev. 2014 (11). PMID: CD000011. doi: 10.1002/14651858.CD000011.pub4.

8. Mistry N, Keepanasseril A, Wilczynski NL, Nieuwlaat R, Ravall M, Haynes RB. Technology-mediated interventions for enhancing medication adherence. J Am Med Inform Assoc. 2015 Apr;22(e1):e177-93. PMID: 25726568. doi: 10.1093/jamia/ocu047.

9. Gupta S, Bhatia S. Optimizing medication adherence in children with cancer. Current Opinion in Pediatrics. 2017 02;29(1):41-5. PMID: 27798425. doi: <https://dx.doi.org/10.1097/MOP.0000000000000434>.

10. Mougalian SS, Epstein LN, Jhaveri AP, Han G, Abu-Khalaf M, Hofstatter EW, et al. Bidirectional Text Messaging to Monitor Endocrine Therapy Adherence and Patient-Reported Outcomes in Breast Cancer. JCO Clinical Cancer Informatics. 2017 11;1:1-10. PMID: 30657377. doi: <https://dx.doi.org/10.1200/CCI.17.00015>.

11. White T, Larson H, Minnella A, Hochster HS. Metastatic Colorectal Cancer Management with trifluridine/tipiracil. Clinical Journal of Oncology Nursing. 2017 Apr;21(2):E30-E7. PMID: WOS:000401116100001. doi: 10.1188/17.Cjon.E30-e37.

12. Oldenmenger WH, Geerling JI, Mostovaya I, Vissers KCP, de Graeff A, Reyners AKL, et al. A systematic review of the effectiveness of patient-based educational interventions to improve cancer-related pain. Cancer Treatment Reviews. 2018 Feb;63:96-103. PMID: 29272781. doi: <https://dx.doi.org/10.1016/j.ctrv.2017.12.005>.

13. Coyne KD, Trimble KA, Lloyd A, Petrando L, Pentz J, Van Namen K, et al. Interventions to Promote Oral Medication Adherence in the Pediatric Chronic Illness Population: A Systematic Review From the Children's Oncology Group. Journal of Pediatric Oncology Nursing. 2019 May/Jun;36(3):219-35. PMID: 30943831. doi: <https://dx.doi.org/10.1177/1043454219835451>.

14. Cruz F, Vilela RA, Ferreira EB, Melo NS, Reis P. Evidence on the Use of Mobile Apps During the Treatment of Breast Cancer: Systematic Review. JMIR Mhealth Uhealth. 2019 08 27;7(8):e13245. PMID: 31456578. doi: <https://dx.doi.org/10.2196/13245>.

15. Dalton M, Holzman E, Erwin E, Michelen S, Rositch AF, Kumar S, et al. Patient navigation services for cancer care in low-and middle-income countries: A scoping review. Plos One. 2019 Oct;14(10):14. PMID: WOS:000532567300024. doi: 10.1371/journal.pone.0223537.

16. Medeiros KS, Queiroz JF, Monteiro MN, Costa WA, Cobucci RN, Stransky B, et al. Impact of mobile applications on adherence to cancer treatment: A systematic review and meta-analysis protocol. BMJ Open. 2019;9(11). PMID: 629783618. doi: <http://dx.doi.org/10.1136/bmjopen-2018-027246>.

17. Er CCW, Theng LB, Al Mahmud A, Tsun MTK. A Survey of Digital Health Interventions for Children with Cancer. Int J Serious Games. 2020 Jun;7(2):71-88. PMID: WOS:000541823600005. doi: 10.17083/ijsg.v7i2.340.

18. Konstantinou P, Kassianos AP, Georgiou G, Panayides A, Papageorgiou A, Almas I, et al. Barriers, facilitators, and interventions for medication adherence across chronic conditions with the highest non-Adherence rates: A scoping review with recommendations for intervention development. Transl Behav Med. 2020 01 Dec;10(6):1390-8. PMID: 2010890676. doi: <http://dx.doi.org/10.1093/tbm/ibaa118>.

19. Lopez-Olivo MA, Maki KG, Choi NJ, Hoffman RM, Shih Y-CT, Lowenstein LM, et al. Patient Adherence to Screening for Lung Cancer in the US: A Systematic Review and Meta-analysis. JAMA Network Open. 2020;3(11):e2025102-e. PMID: 147099356. Language: English. Entry Date: 20201126. Revision Date: 20201216. Publication Type: Article. doi: 10.1001/jamanetworkopen.2020.25102.

20. Mohamed MR, Ramsdale E, Loh KP, Arastu A, Xu HW, Obrecht S, et al. Associations of Polypharmacy and Inappropriate Medications with Adverse Outcomes in Older Adults with Cancer: A Systematic Review and Meta-Analysis. Oncologist. 2020 Jan;25(1):E94-E108. PMID: WOS:000488171700001. doi: 10.1634/theoncologist.2019-0406.

21. Murphy M, Bennett K, Hughes CM, Lavan A, Cadogan CA. Interventions to optimise medication prescribing and adherence in older people with cancer: A systematic scoping review. Research in Social & Administrative Pharmacy. 2020 Dec;16(12):1627-31. PMID: WOS:000594634100001. doi: 10.1016/j.sapharm.2020.02.021.

22. Rawther SCH, Pai MS, Fernandes DJ, Mathew S, Chakrabarty J, Devi ES. Specialist nurse initiated interventions in breast cancer care: A systematic review of randomised controlled trials. Journal of Clinical Nursing. 2020 Jul;29(13-14):2161-80. PMID: WOS:000525883100001. doi: 10.1111/jocn.15268.
