# Supplementary material for: Investigation of Intervention Solutions to Enhance Adherence to Oral Anticancer Medicines in Adults: Overview of Reviews
Source: JMIR Cancer. 2022 Apr 27;8(2):e34833. doi: 10.2196/34833 (PMC9096640; doi:10.2196/34833)
Supplement: Multimedia Appendix 6 [file cancer_v8i2e34833_app6.docx]

Appendix 6. Methodological quality of included systematic reviews (AMSTAR 2 critical domains, adapted from Shea, Reeves [1])

| Critical domains | 2. Protocol | 4. Comprehensive search strategy | 7. Details of excluded studies and justification | 9. Risk of bias (RoB) assessment | 11. Statistical combination of results | 13. Implication of RoB in primary studies | 15. Publication bias | Rating overall confidence in the results of the review |
| --- | --- | --- | --- | --- | --- | --- | --- | --- |
| Mathes 2014 [2] | No | Yes | No | Yes | NA | Yes | NA | CRITICALLY LOW |
| Arthurs 2015 [3] | Partial Yes | Yes | Yes | Yes | NA | Yes | NA | MODERATE |
| Kavookjian 2015 [4] | No | Yes | No | Yes | NA | Yes | NA | CRITICALLY LOW |
| Robertson 2015 [5] | No | Yes | No | Yes | NA | Yes | NA | CRITICALLY LOW |
| Greer 2016 [6] | Partial Yes | No | No | Yes | NA | Yes | NA | CRITICALLY LOW |
| Hurtado-de-Mendoza 2016 [7] | No | No | No | Yes | NA | Yes | NA | CRITICALLY LOW |
| Colombo 2017 [8] | Partial Yes | Yes | Yes | No | NA | Yes | NA | LOW |
| Zerillo 2018 [9] | Partial Yes | Yes | No | Yes | NA | Yes | NA | LOW |
| Ekinci 2018 [10] | No | Yes | No | No | NA | No | NA | CRITICALLY LOW |
| Finitsis 2019 [11] | No | Yes | Yes | Yes | Yes | Yes | Yes | LOW |
| Heiney 2019 [12] | No | No | No | Yes | NA | Yes | NA | CRITICALLY LOW |
| Ruiz-Perez 2019 [13] | Yes | Yes | No | Yes | NA | Yes | NA | LOW |

NA: Not Applicable

References

[1] Shea BJ, Reeves BC, Wells G, Thuku M, Hamel C, Moran J, et al. AMSTAR 2: a critical appraisal tool for systematic reviews that include randomised or non-randomised studies of healthcare interventions, or both. Bmj. 2017;358:j4008-j. 10.1136/bmj.j4008.

[2] Mathes T, Antoine S-L, Pieper D, Eikermann M. Adherence enhancing interventions for oral anticancer agents: a systematic review. Cancer Treatment Reviews. 2014;40:102-8. 10.1016/j.ctrv.2013.07.004.

[3] Arthurs G, Simpson J, Brown A, Kyaw O, Shyrier S, Concert CM. The effectiveness of therapeutic patient education on adherence to oral anti-cancer medicines in adult cancer patients in ambulatory care settings: a systematic review. JBI Database Of Systematic Reviews And Implementation Reports. 2015;13:244-92. <https://dx.doi.org/10.11124/jbisrir-2015-2057>.

[4] Kavookjian J, Wittayanukorn S. Interventions for adherence with oral chemotherapy in hematological malignancies: A systematic review. Research in Social & Administrative Pharmacy. 2015;11:303-14. 10.1016/j.sapharm.2014.08.006.

[5] Robertson EG, Wakefield CE, Marshall KH, Sansom-Daly UM. Strategies to improve adherence to treatment in adolescents and young adults with cancer: a systematic review. Clin Oncol Adolesc Young Adults. 2015;5:35-49. 10.2147/coaya.S85988.

[6] Greer JA, Amoyal N, Nisotel L, Fishbein JN, MacDonald J, Stagl J, et al. A Systematic Review of Adherence to Oral Antineoplastic Therapies. Oncologist. 2016;21:354-76. <https://dx.doi.org/10.1634/theoncologist.2015-0405>.

[7] Hurtado-de-Mendoza A, Cabling ML, Lobo T, Dash C, Sheppard VB. Behavioral Interventions to Enhance Adherence to Hormone Therapy in Breast Cancer Survivors: A Systematic Literature Review. Clin Breast Cancer. 2016;16:247-55.e3. <http://dx.doi.org/10.1016/j.clbc.2016.03.006>.

[8] Colombo LRP, Aguiar PM, Lima TM, Storpirtis S. The effects of pharmacist interventions on adult outpatients with cancer: A systematic review. J Clin Pharm Ther. 2017;42:414-24. <https://dx.doi.org/10.1111/jcpt.12562>.

[9] Zerillo J, Goldenberg B, Kotecha R, Tewari A, Jacobson J, Krzyzanowska M. Interventions to Improve Oral Chemotherapy Safety and Quality: A Systematic Review. JAMA Oncol 2018;4:105-17. 10.1001/jamaoncol.2017.0625.

[10] Ekinci E, Nathoo S, Korattyil T, Vadhariya A, Zaghloul HA, Niravath PA, et al. Interventions to improve endocrine therapy adherence in breast cancer survivors: what is the evidence? Journal of Cancer Survivorship. 2018;12:348-56. <https://dx.doi.org/10.1007/s11764-017-0674-4>.

[11] Finitsis DJ, Vose BA, Mahalak JG, Salner AL. Interventions to promote adherence to endocrine therapy among breast cancer survivors: A meta-analysis. Psycho-Oncology. 2019;28:255-63. <https://dx.doi.org/10.1002/pon.4959>.

[12] Heiney SP, Parker PD, Felder TM, Adams SA, Omofuma OO, Hulett JM. A systematic review of interventions to improve adherence to endocrine therapy. Breast Cancer Research and Treatment. 2019;173:499-510. <http://dx.doi.org/10.1007/s10549-018-5012-7>.

[13] Ruiz‐Pérez I, Rodríguez‐Gómez M, Pastor‐Moreno G, Escribá‐Agüir V, Petrova D. Effectiveness of interventions to improve cancer treatment and follow‐up care in socially disadvantaged groups. Psycho-Oncology. 2019;28:665-74. 10.1002/pon.5011.
