# Supplementary material for: Investigation of Intervention Solutions to Enhance Adherence to Oral Anticancer Medicines in Adults: Overview of Reviews
Source: JMIR Cancer. 2022 Apr 27;8(2):e34833. doi: 10.2196/34833 (PMC9096640; doi:10.2196/34833)
Supplement: Multimedia Appendix 7 [file cancer_v8i2e34833_app7.docx]

Appendix 7. Methodological quality of included systematic reviews (AMSTAR 2 all domains)

| Systematic reviews | 1.PICO components | 2. Protocol | 3. Study design explanation | 4. Comprehensive search strategy | 5. Duplicate study selection | 6. Duplicate data extraction | 7. Details of excluded studies and justification | 8. Description of included studies | 9. Risk of bias (RoB) assessment | 10. Funding sources of included studies | 11. Statistical combination of results | 12. Impact of RoB on meta-analysis results | 13. Implication of RoB in primary studies | 14. Discussion of heterogeneity | 15. Publication bias | 16. Conflict of interest |
| --- | --- | --- | --- | --- | --- | --- | --- | --- | --- | --- | --- | --- | --- | --- | --- | --- |
| Mathes 2014 | Yes | No | No | Yes | Yes | No | No | Yes | Yes | Yes | NA | NA | Yes | Yes | NA | Yes |
| Arthurs 2015 | Yes | Partial Yes | Yes | Yes | No | Yes | Yes | Yes | Yes | No | NA | NA | Yes | Yes | NA | Yes |
| Kavookjian 2015 | Yes | No | Yes | Yes | Yes | No | No | Yes | Yes | No | NA | NA | Yes | Yes | NA | No |
| Robertson 2015 | Yes | No | Yes | Yes | Yes | Yes | No | Yes | Yes | Yes | NA | NA | Yes | Yes | NA | Yes |
| Greer 2016 | Yes | Partial Yes | Yes | No | Yes | No | No | Yes | Yes | Yes | NA | NA | Yes | Yes | NA | Yes |
| Hurtado-de-Mendoza 2016 | Yes | No | Yes | No | Yes | Yes | No | Yes | Yes | No | NA | NA | Yes | Yes | NA | Yes |
| Colombo 2017 | Yes | Partial Yes | Yes | Yes | Yes | Yes | Yes | Yes | No | No | NA | NA | Yes | Yes | NA | Yes |
| Zerillo 2018 | Yes | Partial Yes | No | Yes | Yes | Yes | No | Yes | Yes | No | NA | NA | Yes | No | NA | No |
| Ekinci 2018 | Yes | No | No | Yes | No | No | No | Yes | No | No | NA | NA | No | No | NA | Yes |
| Finitsis 2019 | Yes | No | Yes | Yes | No | Yes | Yes | Yes | Yes | No | Yes | Yes | Yes | Yes | Yes | Yes |
| Heiney 2019 | Yes | No | Yes | No | Yes | No | No | Yes | Yes | Yes | NA | NA | Yes | Yes | NA | No |
| Ruiz-Perez 2019 | Yes | Yes | No | Yes | No | Yes | No | Yes | Yes | Yes | NA | NA | Yes | Yes | NA | Yes |

NA: Not Applicable
